# LncRNAs and their RBPs: How to influence the fate of stem cells?

**DOI:** 10.1186/s13287-022-02851-x

**Published:** 2022-05-03

**Authors:** Cong Zhao, Wen Xie, Hecheng Zhu, Ming Zhao, Weidong Liu, Zhaoping Wu, Lei Wang, Bin Zhu, Shasha Li, Yao Zhou, Xingjun Jiang, Qiang Xu, Caiping Ren

**Affiliations:** 1 Cancer Research Institute, Department of Neurosurgery, National Clinical Research Center for Geriatric Disorders, Xiangya Hospital, Central South University, Changsha, 410008 China; 2 Changsha Kexin Cancer Hospital, Changsha, 410205 China; 3 The Key Laboratory of Carcinogenesis of the Chinese Ministry of Health and the Key Laboratory of Carcinogenesis and Cancer Invasion of the Chinese Ministry of Education, School of Basic Medicine, Central South University, Changsha, 410008 China; 4 Department of Neurosurgery, Xiangya Hospital, Central South University, Changsha, 410008 China; 5 Department of Orthopedics, The Affiliated Zhuzhou Hospital of Xiangya Medical College, Central South University, Zhuzhou, 412007 China; 6 School of Materials Science and Engineering, Central South University, Changsha, 410083 China

**Keywords:** LncRNAs, RBPs, Stem cells, Self-renewal, Differentiation

## Abstract

Stem cells are distinctive cells that have self-renewal potential and unique ability to differentiate into multiple functional cells. Stem cell is a frontier field of life science research and has always been a hot spot in biomedical research. Recent studies have shown that long non-coding RNAs (lncRNAs) have irreplaceable roles in stem cell self-renewal and differentiation. LncRNAs play crucial roles in stem cells through a variety of regulatory mechanisms, including the recruitment of RNA-binding proteins (RBPs) to affect the stability of their mRNAs or the expression of downstream genes. RBPs interact with different RNAs to regulate gene expression at transcriptional and post-transcriptional levels and play important roles in determining the fate of stem cells. In this review, the functions of lncRNAs and their RBPs in self-renewal and differentiation of stem cell are summarized. We focus on the four regulatory mechanisms by which lncRNAs and their RBPs are involved in epigenetic regulation, signaling pathway regulation, splicing, mRNA stability and subcellular localization and further discuss other noncoding RNAs (ncRNAs) and their RBPs in the fate of stem cells. This work provides a more comprehensive understanding of the roles of lncRNAs in determining the fate of stem cells, and a further understanding of their regulatory mechanisms will provide a theoretical basis for the development of clinical regenerative medicine.

## Introduction

Stem cells have the unique abilities to perpetuate themselves through self-renewal and to generate at least one type of highly mature cells through differentiation. According to their sources, stem cells can be divided into embryonic stem cells (ESCs), adult stem cells (ASCs) and induced pluripotent stem cells (iPSCs) [1]. Adult stem cells mainly include neural stem cells (NSCs), mesenchymal stem cells (MSCs), hematopoietic stem cells (HSCs), reproductive stem cells (RSCs), etc. [2]. iPSCs, which reduce ethical disputes in clinical practice, are the most commonly used cell lines in stem cell research [3]. According to different differentiation potentials, stem cells can be divided into totipotent stem cells (TSCs), pluripotent stem cells (PSCs), multipotent stem cells (MSCs) and unipotent stem cells (USCs) [4]. In recent years, the research of stem cell has made remarkable progresses. Because stem cells have the potential for multidirectional differentiation and self-renewal ability, they play important roles in repairing and replacing damaged cells, tissues and even organs, making them important seed cells for regenerative medicine at present and in the future. The application of stem cell depends on a thorough uncovering of the regulatory mechanisms involved in their cellular activities. For example, the transcriptional networks regulated by key transcription factors, such as Oct4, Sox2, and Nanog, have essential roles in maintaining pluripotency of stem cells [5–8]. Besides stem cell related proteins, many studies have identified that multiple species of RNAs are involved in the regulation of stem cell fate. Chepelev and Chen summarized a distinct splicing pattern through which stem cells can be maintained by specific mRNA isoforms, while they lead to proper differentiation after switching to other different isoforms [9]. There are also different RNAs that play important roles in precisely regulating the self-renewal of muscle stem cells [10, 11]. Pereira et al. observed that precise RNA degradation machinery is closely related to stem cell development [12]. Belair et al. demonstrated that exosomes can regulate human ESC (hESC) differentiation and RNA decay plays an importance in maintaining pluripotency [13]. LncRNA is a kind of transcript more than 200 nucleotides, which lacks protein coding potential and plays a regulatory role in maintaining the functions of stem cells. LncRNAs can form a complex with one or more RBPs and play important roles in the development of life. This review summarizes the functions of lncRNAs and their RBPs in stem cell self-renewal and differentiation.

## Functions of long noncoding RNA in stem cells

**Fig. 1:**
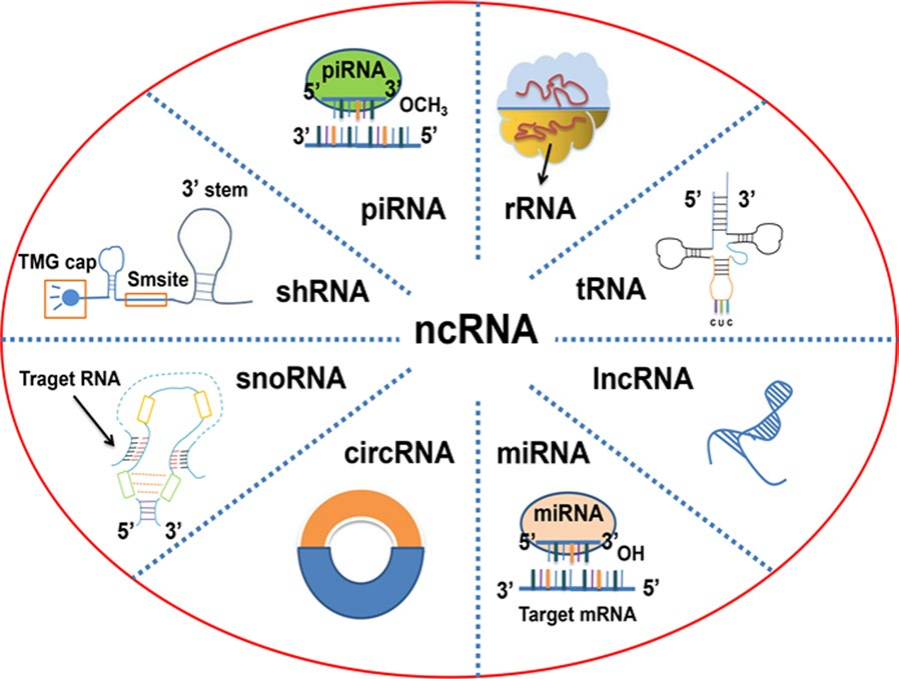
Schematic diagram of different ncRNA structures. The figure shows the classification of noncoding RNA and their structure, including piwi-interacting RNA (piRNA), short hairpin RNA (shRNA), small nucleolar RNA (snoRNA), circular RNA (circRNA), microRNA (miRNA), long noncoding RNA (lncRNA), transfer RNA (tRNA), ribosomal RNA (rRNA)

RNA is a carrier of genetic information that exists in organisms and certain viruses. It performs the expression of genetic information encoded by DNA on proteins and controls various biological processes of organisms [14]. According to their function, RNAs can be divided into coding RNAs and ncRNAs. Coding RNAs generally refer to mRNA that encodes protein. ncRNAs are transcribed from DNA, which do not encode proteins. ncRNAs can be divided into the following two types according to their length: small ncRNA and long ncRNA [15]. Small ncRNAs refer to ncRNAs shorter than 200 nucleotides, while long ncRNAs (lncRNAs) are composed of 200 or more nucleotides. According to their function and subcellular location, small ncRNAs can be divided into microRNAs (miRNAs), transfer RNAs (tRNAs), piwi-interacting RNAs (piRNAs), small nucleolar RNAs (snoRNAs), short hairpin RNAs (shRNAs), circular RNAs (circRNAs), ribosomal RNAs (rRNAs), etc. [16, 17] (Fig. 1).

As a complex regulator in many biological processes, noncoding RNA forms an important part of the stem cell transcriptome, which is essential for the self-renewal and pluripotency maintenance of stem cells. Many studies have found that different types of ncRNAs, including lncRNAs [18, 19], microRNAs [20, 21], circRNAs [22], etc., have important influences on the fate of stem cells and may participate in different biological processes of stem cells, such as proliferation, apoptosis and differentiation. LncRNA growth arrest specific 5 (Gas5) is a host gene of small nucleolar RNA, involved in many biological functions of mESCs. Down-regulation of Gas5 expression can reduce the efficiency of somatic cell reprogramming [23]. LncRNAs are vital to the self-renewal and pluripotency maintenance of stem cells through different regulatory mechanisms. They take part in stem cell activities through regulatory mechanisms such as chromatin or histone modification around transcription factor genes, typical and atypical RNA-binding proteins, and as a sponge for microRNAs [24]. In general, lncRNAs can serve as miRNA sponge or competitive endogenous RNAs (ceRNAs) in stem cells [25]. Cyrano is a lncRNA located in the cytoplasm and nucleus of ESC, and it is a part of ncRNA regulatory network [26]. Cyrano silencing can inhibit the self-renewal and survival of ESCs, and its direct interaction with miR-7 can enhance the expression of the core pluripotency regulator Nanog to promote self-renewal of ESCs [27]. According to previous reports, lncRNAs are new regulators in the osteogenesis of MSCs [28]. The enhancement of special AT-rich sequence-binding protein 2 (SATB2) can promote the osteogenic differentiation of bone marrow mesenchymal stem cells (BMSCs) when patients have ethanol-induced osteonecrosis [29, 30]. The exosomes secreted by BMSCs contain lncRNA metastasis-associated lung adenocarcinoma transcript 1 (MALAT1), which can act as a sponge for miR-34c, increase the expression of SATB2 and help alleviate osteoporosis [31]. Wang et al. confirmed that lncRNA-ROR stabilizes the expression of the main pluripotent genes Oct4, Nanog and Sox2 by inhibiting miR-145, maintaining the pluripotency of hESCs [32].

## Functions of RNA-binding protein in stem cells

RNA-binding proteins are a group of proteins involved in RNA-related metabolic regulatory processes. They can bind double or single-stranded RNAs to participate in the formation of ribonucleoprotein (RNP) complexes and influence the fate of RNA. RBPs, as trans-acting factors, are mainly mediated by specific RNA-binding domains (RBDs) [33]. They affect all aspects of post-transcriptional regulation and act in the regulation of RNA synthesis, splicing, modification, nuclear output, localization, protein stability and translation rate [34]. These RBPs functions are highly dependent on their structural characteristics, namely the presence and alignment of RNA-binding domains. Most RBPs contain multiple RNA-binding domains and RNA-binding motifs, which can recruit multiple RNAs to bind to them. Therefore, studying the interaction between RNA and protein is critical to exploring the function of RNA [35]. There is abundant research evidence that RBP plays an important role in stem cells. Most lncRNAs interact with the corresponding RBPs [36] and play irreplaceable roles in various cells and physiological processes. The interaction of different lncRNAs and their RBPs is responsible for a large number of biological events in stem cells, such as changing or stabilizing their nuclear outlet, regulating transcription or translation, especially playing an important role in stem cell self-renewal and maintenance of pluripotency [37, 38].

## The functions of lncRNAs and their RBPs in stem cells

**Fig. 2:**
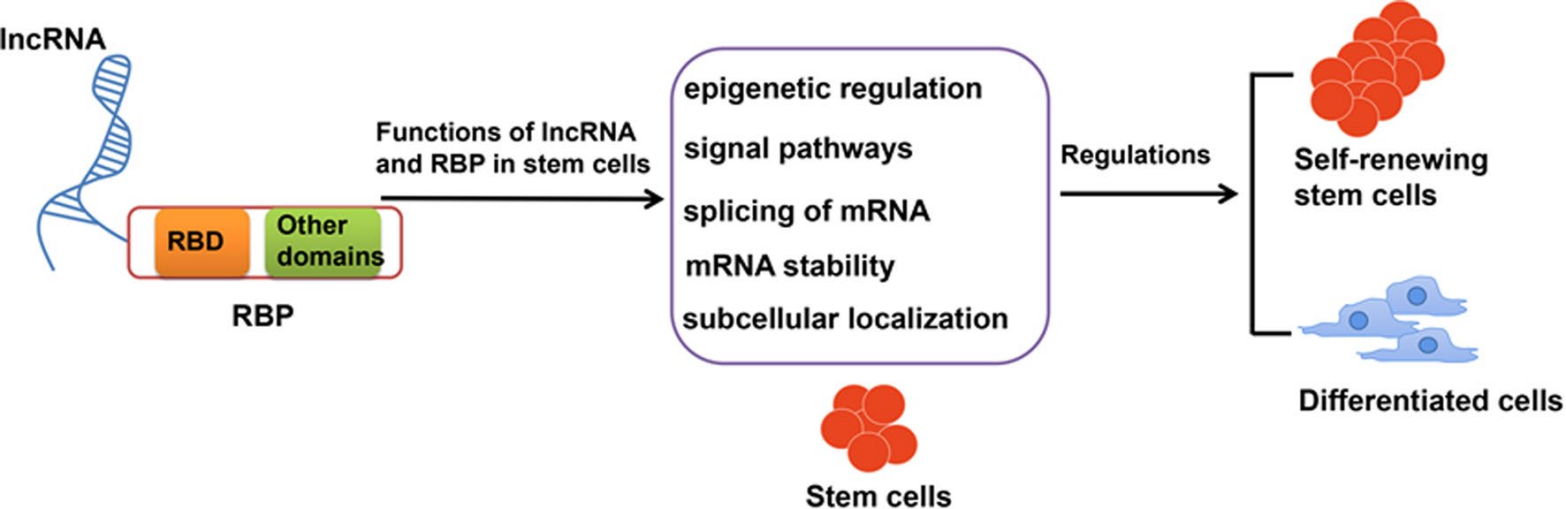
Functional crosstalk between lncRNAs and their RBPs. LncRNA binds to RBP to control the self-renewal and differentiation of stem cells through five regulatory mechanisms, including epigenetic regulation, signal pathways, splicing of mRNA, mRNA stability and subcellular localization

LncRNAs and RBPs are key regulators of gene expression and the core of many cells functions, such as protein synthesis, mRNA assembly, virus replication, and cells development regulation. Together, they have a profound effect on life activities of stem cells [39]. LncRNAs usually function in stem cell by binding RBPs. LncRNA Cyrano binds to signal transducer and activator of transcription 3 (STAT3), which is essential for the proliferation and self-renewal of ESCs [40]. Zhao et al. identified 39 lncRNAs binding to Klf4 by RNA immunoprecipitation and sequencing (RIP-SEQ) assay in the process of studying the role of lncRNA sites in tissue 3D chromatin structure. Considering the known role of lncRNAs in maintenance and inducing pluripotency, lncRNA 5430416N02Rik was screened. The interaction between 5430416N02Rik and Mid1 loci activates Mid1 expression and promotes Mid1 transcription, which in turn promotes the rapid proliferation of ESCs [41]. LncRNAs have been proved to play an important role in the osteogenic differentiation of MSC. Periodontal ligament stem cells (PDLSCs) are MSCs derived from oral tissues and possess multi-dimensional differentiation potential and good self-renewal ability. Osteogenic differentiation is one of the most important characteristics of oral stem cell pluripotency, which plays a significant role in periodontal tissue regeneration [42]. Lin28 is a key factor regulating development time. Lin28 has two conserved paralogs, lin28 homolog A (lin28A) and lin28 homolog B (lin28B) [43]. Previous studies have found that lin28A is involved in cells development, metabolism and stem cell maintenance [44, 45]. LncRNA taurine up-regulated gene 1 (TUG1) is mainly distributed in the nucleus of PDLSCs, and the dynamic expression of TUG1 is increased in osteogenic PDLSCs. As a regulator of PDLSCs osteogenesis, TUG1 interacts with lin28A to activate lin28A and promote the expression of multiple osteogenic genes, thereby promoting the osteogenic differentiation of PDLSCs [46]. In this review, we elaborate the specific regulatory mechanism of lncRNAs and their RBPs in regulating the fate of stem cells, which is of great significance to explore the therapeutic potential of stem cells in medicine. The functions of lncRNAs and their RBPs mainly include the following five aspects. Next, we will focus on these five regulatory mechanisms and illustrate the functions of lncRNAs and their RBPs in stem cells (Fig. 2).

### LncRNAs and their RBPs affect epigenetic regulation in stem cells

Epigenetic regulatory factors are one of the key regulatory factors for cells differentiation. As an emerging epigenetic factor, ncRNAs have an important impact on determining the fate of stem cells. LncRNA-binding proteins can directly regulate or enhance the transcription of gene expression to maintain the state of stem cells. The function of lncRNAs will change with its cell location. Nuclear lncRNAs can recruit chromatin modifying factors and other proteins or transcription factors. The nuclear localization of lncRNAs provides evidence for epigenetic and transcriptional regulation.

Polycomb inhibitory complex 2 (PRC2) is a protein complex that has epigenetic regulation function and can maintain histone modifications [47]. The mammalian PRC2 core complex includes enhancer of zeste homolog 1/2 (EZH1 nd/or EZH2), embryonic ectoderm development (EED) and suppressor of zeste 12 (SUZ12). EZH1/2 is the catalytic subunit of PRC2 and each subunit contains a SET domain. Through this domain, the methyl portion of the cofactor s-adenosine-l-methionine (SAM) is transferred to lys27 of histone H3 (H3K27), but EZH2 itself lacks histone methyltransferase (HMTase) activity and requires the assistance of the other two core subunits of PRC2 [48]. H3K27me3 is one of the dominant parts of epigenetic regulation [49]. In human mesenchymal stem cells (hMSCs), inhibition of EZH2 can reduce adipogenesis and induce bone formation [50]. LncRNA maternally expressed 3 (MEG3) can interact with EZH2, and the down-regulation of MEG3 or EZH2 promotes the osteogenic differentiation of human dental follicle stem cells (hDFSCs) by reducing the occupation of H3K27me3 on the Wnt gene promoter [51]. Targeted degradation of EZH2 may have the potential to induce bone formation of bone marrow mesenchymal stem cells, which provides important evidence for the treatment of MSC-related diseases such as bone aging and osteoporosis. Oct4 is expressed in germ cells, the inner cell mass of preimplantation embryos, and embryonic stem cells. Among mouse ESCs (mESCs), Oct4 is essential for establishing a core transcription network that maintains pluripotency and self-renewal. The researchers found that lncRNA Oct4 pseudogene 4 (Oct4P4), which is derived from the mouse Oct4 pseudogene, is an epigenetic regulator of gene expression and has a bearing on the self-renewal of mESC. During mESC differentiation, Oct4P4 is up-regulated and combined with SUV39H1 HMTase to form a complex, so that the complex recruits H3K9me3 and HP1α to the Oct4 promoter region, resulting in Oct4 silencing and prevention of mESC self-renewal [52]. LncRNA ES1, ES2 and ES3, as nuclear lncRNAs, can interact with SUZ12 and Sox2 that are components of the PRC2 complex, to maintain the pluripotency of hESCs [53]. Large-intergenic noncoding RNAs (lincRNAs) 1614 can bind to multiple pluripotency factors, which is essential for maintaining the pluripotency of ESCs. Linc1614 interacts with Sox2 and recruits the PRC2 complex to T, Eomes, and Pitx2 and other developmental gene regions and inhibits the expression of these developmental genes through catalytic inhibition of H3K27me3 modification to maintain the pluripotency of mESCs [54]. p53 is a tumor suppressor that actively participates in the differentiation of hESCs and mESCs. Jain et al. discovered that a p53-regulated, pluripotency-specific lncRNA, p53-regulated and ESC-associated 1 (PRESS1) can interact with sirtuin6 (SIRT6) and inhibit SIRT6 from attaching to chromatin, thus maintaining the acetylation level of Histone H3K56 and H3K9 on promoters of pluripotent genes such as Oct4 and Nanog to protect hESC pluripotency [55]. A new research identified a new type of lncRNA that is expressed at a low level in bladder cancer stem cells (BCSCs). LncRNA LBCS binds to heterogeneous ribonucleoprotein K (hnRNPK) and EZH2 and acts as a scaffold to induce the formation of the hnRNPK-EZH2 complex. Next, LBCS guides the complex to the Sox2 promoter and H3K27me3 to inhibit Sox2 expression and the self-renewal of BCSCs [56]. The discovery of this regulatory mechanism not only indicates that lncRNAs have a significant epigenetic regulation role in the process of tumorigenesis, but also provides potential prognostic indicators and theoretical basis for the treatment of bladder cancer.

### LncRNAs and their RBPs are involved in the regulation of signaling pathways in stem cells

Signaling pathways, including fibroblast growth factor (FGF)/extracellular signal-regulated kinase (ERK) signaling pathway, notch, bone morphogenetic protein (BMP), Wnt and other signaling pathways, are key regulatory mechanisms controlling the functions of various stem cells. These signal pathways can interact with each other to form a synergistic or antagonistic signal network, but most of them are independent of each other. Stem cells generally maintain their pluripotency and self-renewal by activating molecular pathways required for self-renewal and inhibiting genes required for differentiation.

LincU, which is localized in the cytoplasm, is highly expressed in a Nanog-dependent manner in naive stem cells. LincU can stabilize Dusp9 and inhibit MAPK/ERK signal through degradation mediated by ubiquitinated proteasome, enhancing the pluripotency of mESCs [57]. Thoc5 is an essential factor for trim71 interacting long noncoding RNA 1 (Trincrl) nuclear export and an essential component of ESCs to inhibit ERK signaling. Thoc5 promotes the export of Trincr1 to the cytoplasm. In the cytoplasm, lncRNA Trincr1 binds to tripartite motif 71 (Trim71), inhibits the stability of SHC binding and spindle associated 1 (SHCBP1), leads to down-regulation of SHCBP1 protein, phosphorylates ERK and regulates the expression of ERK pathway target genes, and then makes mESCs that depend on Trim71 can proliferate rapidly [58]. The normal differentiation of mESCs is coordinated by transcription factors, epigenetic regulatory factors, chromatin regulatory factors and signaling pathways. According to reports, the leukemia inhibitory factor (LIF)/STAT3 signaling pathway is essential for maintaining the self-renewal and pluripotency of mESCs [59]. Linc1557 is mainly located in the cytoplasm of mESCs and directly interacts with STAT3 through specific binding sites to regulate the stability of its mRNA, regulating the LIF/STAT3 signaling pathway and promoting the self-renewal of mESCs [60]. Peptidylprolyl Isomerase E (PPIE) is an RNA-binding protein located in the nucleus of hESCs [61]. PPIE can regulate lncRNA hFAST processing and export and help it get into cytoplasm. In the cytoplasm of hESCs, the hFAST binds to the WD40 domain of beta-transducin repeat containing E3 ubiquitin protein (β-TrCP) and blocks β-TrCP interaction with phosphorylated β-catenin to prevent its degradation. Thus, Wnt signaling is activated and maintains the pluripotency of hESCs [62]. LncR492 is a lineage-specific inhibitor of neuroectodermal differentiation. Human embryonic lethal abnormal vision-like protein HuR belongs to the Hu family of RNA-binding proteins. Under normal cellular and physiological conditions, HuR is mainly located in the nucleus. When pressure is applied to it, HuR can migrate to the cytoplasm and stabilize and increase the translation of target mRNA [63]. Researchers have discovered that lncR492 interacts with the mRNA-binding protein HuR and inhibits the neuroectodermal differentiation of mESCs by activating Wnt signaling [64]. Not only stem cells have the characteristics of self-renewal and rare differentiation, but cancer stem cells also have the characteristics of stemness. The Switch/Sucrose Nonfermentable (SWI/SNF) complex is a chromatin remodeling complex composed of SNF5, BRG1 and BAF170. LncRNA T cell factor 7 (TCF7) binds to BAF170 and recruits the SWI/SNF complex to the TCF7 promoter to regulate its expression, leading to the activation of Wnt signaling and promoting the self-renewal of liver cancer stem cells (liver CSCs) [65]. The Hippo-Yes-Associated Protein (YAP) signaling pathway mediates the differentiation and self-renewal capabilities of a variety of adult stem cells and affects the adipose osteogenic differentiation of hMSCs [66]. Human dental pulp stem cells (hDPSCs) are considered as MSCs due to their strong ability to proliferate and differentiate into different lineages [67]. LncRNA H19 recruits EZH2 to the large tumor suppressor 1 (LATS1) promoter region to induce H3K27me3, inhibiting the expression of LATS1, blocking the activation of the Hippo-YAP signaling pathway and enhancing the dentin differentiation and proliferation of hDPSCs [68]. BMP signaling pathway is involved in the self-renewal and differentiation process of ESCs [69, 70]. LncRNA heart and neural crest derivatives expressed 2-antisense RNA 1 (HAND2-AS1) combines with INO80 complex to promote the expression of bone morphogenetic protein receptor 1A (BMPR1A) and activate the BMP signal, thus promoting the self-renewal of liver CSCs. HAND2-AS1 promotes the self-renewal of liver CSCs and the occurrence of liver tumors, providing a potential new target for the treatment of hepatocellular carcinoma (HCC) [71].

### LncRNAs and their RBPs regulate the splicing of mRNA in stem cells

Splicing is an mRNA processing event. The splicing of mRNA can be used as a gateway to control the self-renewal and differentiation of stem cells. The coordinated control of mRNA and rRNA processing controls the pluripotency and differentiation of embryonic stem cells [72]. LncRNAs and their RBPs participate in the regulation of stem cell self-renewal and pluripotency maintenance through the splicing of mRNA. Protein kinase C δ (PKCδ) is a factor that can activate the survival and proliferation of neurons. Splicing of PKCδII can increase the survival rate of neurons. The exosomes secreted by human adipose stem cells (hASCs) contain lncRNA MALAT1, and lncRNA MALAT regulates the splicing of genes mainly by regulating the activities of splicing factors. MALAT1 promotes splicing or controls the assembly of complexes that mediate gene expression to regulate the expression of downstream genes. It can be absorbed by hippocampal HT22 cells and promotes the splicing of PKCδII by binding the splicing factor serine-rich arginine splicing factor 2 (SRSF2), which leads to the proliferation of neurons in the brain injury site of hASCs. This holds great promise for the treatment of traumatic brain injury and other neurodegenerative diseases [73]. MEG3 is a kind of lncRNA that can regulate the osteogenic differentiation of MSCs through a variety of ways. Up-regulated MEG3 interacts with hnRNPI to inhibit the expression of BMP2. HnRNPI plays an active role in mRNA splicing and significantly inhibits osteogenic differentiation of human periodontal ligament cells (hPDLCs) through this regulatory mechanism [74]. This discovery also provides a new method for the treatment of periodontitis.

Splicing can occur either cis or trans, where cis splicing connects exons of a single pre-mRNA, while trans-splicing connects exons of two or more independent pre-mRNAs from the same gene (intra-trans-splicing) or two or more different genes (intergene trans-splicing). It usually occurs in single-celled organisms, nematodes, and trypanosomes [75]. Trans-splicing connects different pre-mRNA exons for splicing to generate different transcripts from a limited number of genes. This is a post-transcriptional event. Studies have found that trans-splicing lncRNAs influence on the pluripotency maintenance of stem cells [76, 77]. Trans-spliced noncoding RNA RMST (tsRMST) is a typical example of trans-splicing that occurs in normal human cells and is a trans-splicing subtype of RMST. RMST is a lncRNA gene located on the qarm of chromosome 12, with about 2.6 kb in length, which plays an important role in the process of neural differentiation [54]. The expression of RMST is critical for the binding of Sox2 to neurogenic genes, and tsRMST is an emerging regulatory lncRNA associated with human stem cell pluripotency [54, 78]. Yu et al. demonstrated that tsRMST is highly expressed in hESCs and down-regulated during in vitro differentiation. It interacts with Nanog and SUZ12 to form a complex that inhibits the expression of Wnt5A along with differentiation-related transcription factors and inhibits non-standard Wnt pathways, preventing differentiation of hESCs [79]. tsRMST inhibits gene expression through association with pleiotropic-related transcription factors, PRC2 components and subsequent trimethylation of H3K27 in the target gene promoter region [80]. Furthermore, microarray and Ingenious Pathway Analysis (IPA) have demonstrated the inhibitory effect of tsRMST on noncanonical Wnt signaling pathway. Previously, the regulatory role of lncRNA combined with RBP in some signaling pathways, especially typical Wnt signaling pathways, has been studied a lot, but the regulatory role of lncRNAs in noncanonical Wnt signaling pathway is poorly understood. Wnt proteins are classified into typical proteins and non-standard proteins. Wnt5A and Wnt5B belong to non-standard proteins [81]. A recent study found that the non-standard Wnt5A/Fzd2 pathway plays an important role in prostate cancer [82]. The discovery of the role of tsRMST in non-standard Wnt signaling pathway provides further insights into the role of lncRNA in regulating signaling pathways and provides important insights into the role of trans-splicing lncRNA in the pluripotency maintenance and lineage differentiation of hESCs.

### LncRNAs and their RBPs are involved in the regulation of the stability of mRNA in stem cells

Many studies have emphasized that gene expression is precisely regulated by mRNA stability. mRNA stability largely affects the secondary and tertiary structures of the mRNAs, and the accessibility of various RBPs to the mRNAs. The stability of mRNA is regulated by diverse RNA modifications. To date, hundreds of different RNA modifications have been characterized [83]. However, the mRNA stability is also affected by lncRNAs and their RBPs in stem cells.

Heterogeneous nuclear ribonucleoproteins (hnRNPs) belong to a well-known splicing protein family, which has been widely studied in the field of stem cells, and great achievements have been made in recent years. As typical RNA-binding proteins, hnRNPs effect on mRNA stability and gene transcription [84]. HnRNPs bind to lncRNA and form a complex or directly bind to mRNA in stem cells, affecting the stability of mRNA. As a member of the hnRNP family, polypyrimidine tract binding protein 1 (PTBP1 or hnRNPI) is a multifunctional protein associated with neurogenesis and participates in all steps of RNA biogenesis. Its regulation of mRNA stability and pre-mRNA splicing has been demonstrated in previous studies [85]. Studies on the relationship between energy metabolism and the fate of stem cells have identified Lncenc1 as the first lncRNA that connects self-renewal and energy metabolism in PSCs. HnRNPK binds to the promoter of glycolysis genes and directly regulates the transcription of these genes. Lncenc1, hnRNPK and PTBP1 form a complex that regulates the transcription of glycolysis-related genes to maintain glycolytic activity and promote the self-renewal of naive embryonic stem cells (nESCs) [86, 87]. In addition, PTBP1 can perform different functions by shuttling between the nucleus and cytoplasm [88]. In the nucleus, PTBP1 forms a ribonucleoprotein complex and regulates splicing, polyadenylation and mRNA export. In the cytoplasm, PTBP1 is involved in translation initiation and mRNA stability [89]. Knockdown of PTBP1 can cause premature differentiation and impaired motor behavior of NSC in mouse brain [90]. Pnky is a conserved, neuro-specific nuclear lncRNA that plays a unique role in controlling neurogenesis. Knocking down Pnky can lead to the production of neurons. Pnky and PTBP1 specifically bind to each other, which maintains the stability of their mRNA, inhibits the differentiation of NSCs and affects neuronal development [91, 92]. A new study showed that the lncRNA anti-differentiation noncoding RNA (ANCR) located in cytoplasmic binds to PTBP1, promotes the interaction between PTBP1 and inhibitor of DNA binding 2 (ID2) mRNA, and enhances the stability of ID2 mRNA, resulting in inhibition the differentiation of human adipose-derived mesenchymal stem cells (hAMSCs) into definitive endoderm cells (DECs) [93]. When breast cancer cells are under hypoxic conditions, lncRNA KB-1980E6.3 is necessary to maintain stemness. LncRNA KB-1980E6.3 binds to insulin-like growth factor 2 mRNA-binding protein 1 (IGF2BP1) to form a complex under the induction of HIF-1α to recognize m6A modified c-myc coding region instability determinant (CRD) mRNA and enhance the stability of c-myc mRNA, which significantly promotes the stemness and proliferation of breast cancer stem cells (BCSCs) in hypoxic tumor microenvironment [94].

### LncRNAs and their RBPs are involved in the regulation of the subcellular localization in stem cells

LncRNAs, as a kind of RNA that does not encode protein, have been shown to be distributed in both the nucleus and the cytoplasm. In the process of summarizing the functions of lncRNAs and their RBPs, we found that lncRNAs often combine with their RBPs to achieve their functions, affecting the modification level or localization of RBPs and thus affecting downstream genes. LncRNA Panct1 is a polyadenylation transcription unit located in the nucleus and involved in the maintenance of pluripotency of mESCs. Panct1 can interact with transient octamer binding factor 1 (TOBF1) and regulate its subcellular location. Overexpression of Panct1 leads to higher expression of pluripotency genes such as Oct4, Nanog, Zscan4c, Sox2, and Klf4 and leads to the pluripotency maintenance of mESCs [95]. However, the subcellular localization of lncRNAs can also regulate the fate of stem cells. Subcellular localization of lncRNA FAST is different in hESCs and mESCs. hFAST locates in the cytoplasm, while mFAST locates in nucleus. Guo, et al. certified that cytoplasmic hFAST but not nuclear mFAST promotes Wnt signaling in pluripotency maintenance of hESCs. In the cytoplasm of hESCs, the hFAST binds to WD40 domain of β- TrCP, impedes the interaction between β- TrCP and phosphorylated β-catenin and promotes Wnt activity to maintaining pluripotency of hESCs [63].

To date, the regulatory mechanisms underlying the regulation of protein localization and transformation have been rarely studied in embryonic stem cells and adult stem cells, but widely studied in tumor cells. OLA1P2 is an up-regulated lncRNA induced by aspirin. Aspirin can induce the up-regulation of forkhead box D3 (FOXD3) gene expression in tumor cells. FOXD3 protein transcriptionally up-regulates the expression of OLA1P2. Highly expressed OLA1P2 directly binds to phosphorylated STAT3 (Tyr705) protein, which inhibits its nuclear import to a large extent, blocks the formation of phosphorylated STAT3 homodimers, and significantly affects STAT3 signaling, which inhibits the proliferation and migration of cancer cells. The discovery of this regulatory mechanism provides a potential therapeutic target and new insights for cancer chemotherapy [96]. Furthermore, previous studies have shown that FOXD3 is essential for the self-renewal, survival and pluripotency maintenance of human embryonic stem cells. Studying the regulatory mechanism of interaction between OLA1P2 and phosphorylated STAT3 protein may have great value in stem cells [97]. Linc00460 is located in the cytoplasm, hnRNPK is distributed in both cytoplasm and nucleus. Linc00460 binds with and translocates hnRNPK that is located in nucleus to the cytoplasm to participate in special mRNA stability and translation regulation, and promotes cell migration and invasion through inducing EMT in lung cancer [98]. The regulation of lncRNA on the tumor suppressor p53 pathway has been a topic of great interest. Ras-GTase-activating protein SH3 domain binding protein 1 (G3BP1) is a specific binding protein of the SH3 domain of Ras GTPase activating protein (Ras-GAP), a negative feedback regulator of Ras activity. It is distributed in the cytoplasm and has an RRM domain, which can directly interact with RNA, so G3BP1 belongs to the RBP family [99]. Inactivation of p53 is a key event in tumor Linc00460 that is located in the cytoplasm, hnRNPK is distributed in both cytoplasm and nucleus. Linc00460 binds with and translocates hnRNPK that is located in nucleus to the cytoplasm to participate in special mRNA stability and translation regulation, and promotes cell migration and invasion through inducing EMT in lung cancer formation. Mutations, transcription inactivation, abnormal degradation and changes in subcellular localization of p53 may lead to the failure of p53 to perform normal functions. It has been reported that G3BP1 can bind to the RRM domain of p53 and transfer p53 from the nucleus to the cytoplasm. Thus, p53 cannot play its function in the nucleus and promotes the occurrence and development of malignant tumors [100]. However, Mao et al. demonstrated that p53 can return from the cytoplasm to the nucleus and act as a transcription factor to inhibit the proliferation of lung adenocarcinoma cells. The main reason is that lncRNA P53RRA competitively inhibits the binding of G3BP1 and p53 in the cytoplasm through specific binding with G3BP1 [101]. The discovery of this regulatory mechanism will provide a great reference value for subsequent research on self-renewal and pluripotency maintenance of stem cells.

## Conclusions and perspectives

**Fig. 3:**
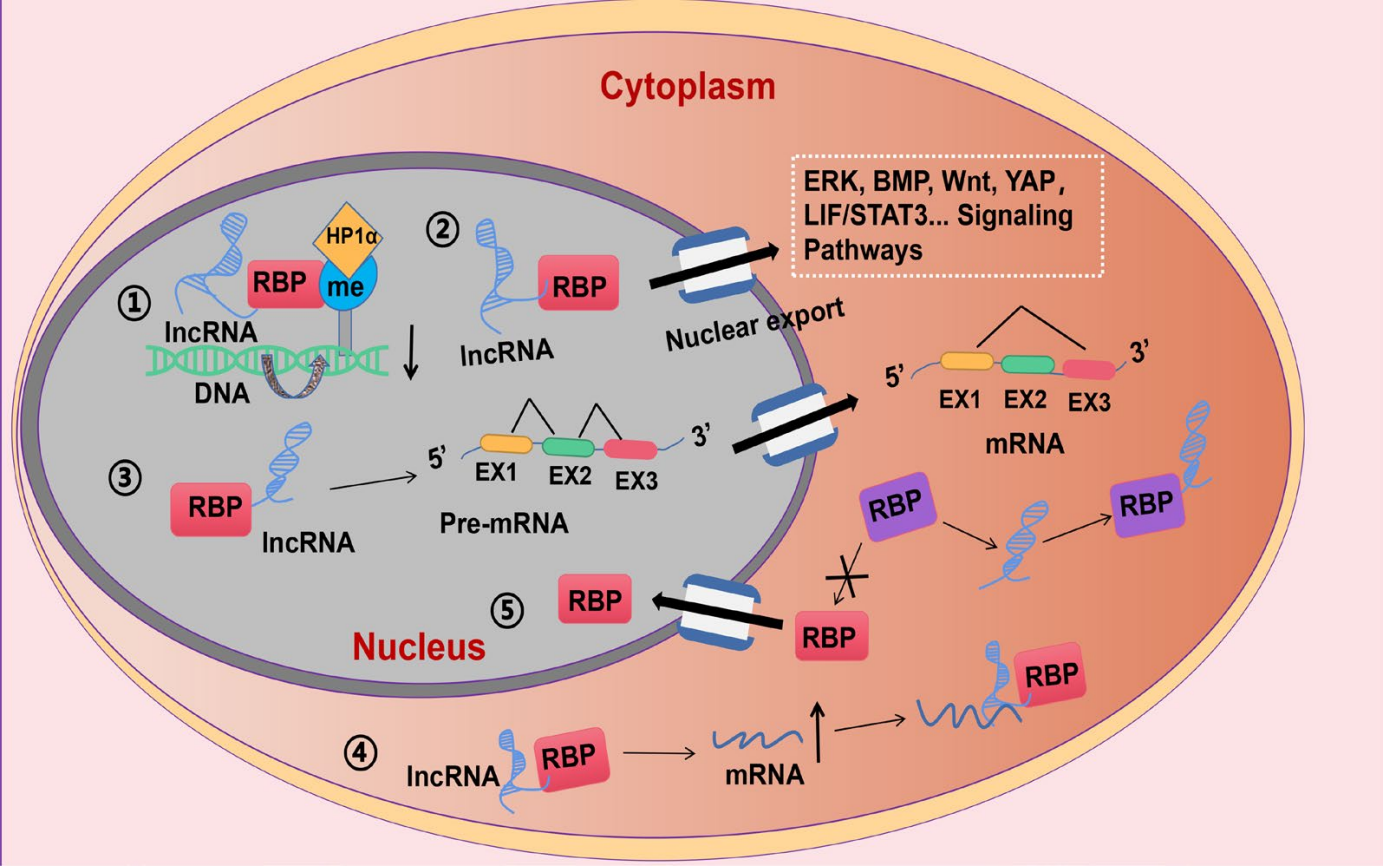
Functions of lncRNAs and their RBPs in self-renewal and differentiation of stem cells. (1) LncRNAs and their RBPs affect epigenetic regulation in stem cells. For example, up-regulated Oct4P4 combines with SUV39H1 HMTase to form a complex, which recruits H3K9me3 and HP1α to the Oct4 promoter region, resulting in Oct4 silencing and preventing mESC self-renewal. (2) LncRNAs and their RBPs are involved in the regulation of signaling pathways in stem cells. For example, lncR492 interacts with the mRNA-binding protein HuR and inhibits the neuroectodermal differentiation of mESCs by activating Wnt signaling. (3) LncRNAs and their RBPs regulate the splicing of mRNA in stem cells. For example, MALAT1 can be absorbed by hippocampal HT22 cells and promotes the splicing of PKCδII by binding the splicing factor SRSF2, which leads to the proliferation of neurons in the brain injury site of hASCs. (4) LncRNAs and their RBPs are involved in the regulation of the stability of mRNA in stem cells. For example, lncRNA ANCR binds to PTBP1, promotes the interaction between PTBP1 and inhibitor of ID2 mRNA and enhances the stability of ID2 mRNA, resulting in inhibition of the differentiation of hAMSCs into DE cells. (5) LncRNAs and their RBPs are involved in the regulation of the subcellular localization in stem cells. For example, lncRNA P53RRA inhibits the binding of G3BP1 and p53 in cytoplasm through specifically binding with G3BP1, so p53 is transferred from cytoplasm to nucleus to function as a transcription factor

There are a large number of lncRNAs in the mammalian genome, which constitute an important part of the genome. RBPs and lncRNAs can affect the localization, stability and translation of target mRNAs. The combination of versatility and structural flexibility between the two enables RBPs and lncRNAs to form a strong regulatory network that is closely related to the function of stem cells [102]. In this review, we focus on the functions by which lncRNAs and their RBPs maintain the self-renewal and pluripotency of stem cells. The main regulatory mechanisms include (1) LncRNAs and their RBPs affect epigenetic regulation in stem cells. (2) LncRNAs and their RBPs are involved in the regulation of signaling pathways in stem cells. (3) LncRNAs and their RBPs regulate the splicing of mRNA in stem cells. (4) LncRNAs and their RBPs are involved in the regulation of the stability of mRNA in stem cells. (5) LncRNAs and their RBPs are involved in the regulation of the subcellular localization in stem cells (Fig. 3, Table 1).

**Table 1:** Functions of LncRNAs and their RBPs in self-renewal and differentiation of stem cells

LncRNA	RBPs	Cell type	Biological function	Regulatory mechanism	References
MEG3	EZH2	hDFSC	Promote the osteogenic differentiation of hDFSC	MEG3 interacts with EZH2, down-regulation of MEG3 or EZH2 reduces the occupation of H3K27me3 on the Wnt gene promoter	[51]
Oct4P4	SUV39H1 HMTase	mESC	Inhibit the self-renewal of mESC	OctP4 combines with SUV39, H1 and HMTase to form a complex, recruits H3K9me3 and HP1α to the Oct4 promoter region and results in Oct4 silencing	[52]
ES1, ES2, ES3	SUZ12 and Sox2	hESC	Maintain the pluripotency of hESC	ES1, ES2 and ES3 interact with SUZ12 and Sox2, which are the components of PRC2 complex	[53]
Linc1614	PRC2 complex and Sox2	mESC	Maintain the pluripotency of mESC	Linc1614 interacts with Sox2, recruits the PRC2 complex to T, Eomes, and Pitx2 and other developmental gene regions and inhibits their expression	[54]
LncPRESS1	SIRT6	mESC	Maintain the pluripotency of mESC	LncPRESS1 interacts with SIRT6 and inhibits SIRT6 from attaching to chromatin, maintaining the acetylation level of Histone H3K56 and H3K9 on the promoters of pluripotent genes such as Oct4 and Nanog	[55]
LBCS	hnRNPK and EZH2	BCSC	Inhibit the self-renewal of BCSC	LBCS binds hnRNPK and EZH2 to form the hnRNPK-EZH2 complex, guides the complex to the Sox2 promoter and H3K27me3 to inhibit Sox2 expression	[56]
LincU	Dusp9	mESC	Maintain the pluripotency of mESC	LincU binds and stabilizes ERK-specific phosphatase DUSP to restrict MAPK/ERK activity	[57]
Trincr1	Trim71	mESC	Promote the proliferation of mESC	Thoc5 regulates the export of Trincr1 to the cytoplasm, lncRNA Trincr1 binds to Trim71 in the cytoplasm, inhibits the activity of SHCBP1 and phosphorylates ERK and promotes the expression of ERK pathway target genes	[58]
Linc1557	STAT3	mESC	Promote the self-renewal of mESC	Linc1557 interacts with STAT3 through specific binding sites to regulate the stability of its mRNA, thus regulating the LIF/STAT3 signaling pathway	[60]
hFAST	β- TrCP	hESC	Maintain the pluripotency of hESC	hFAST binds to WD40 domain of β- TrCP, impedes the interaction between β- TrCP and phosphorylated β-catenin and promotes Wnt activity	[62]
LncR492	HuR	mESC	Inhibit the neuroectodermal differentiation of mESC	LncR492 interacts with HuR and activates Wnt signaling	[64]
TCF7	BAF170 and SWI/SNF complex	liver CSC	Promote the self-renewal of liver CSC	TCF binds to BAF170 and recruits the SWI/SNF complex to the TCF7 promoter to regulate its expression, leading to the activation of Wnt signaling	[65]
H19	EZH2	hDPSC	Promote the dentin differentiation and proliferation of hDPSC	H19 recruits EZH2 to the LATS1 promoter region to induce H3K27me3, inhibiting the expression of LATS1, blocking the activation of the Hippo-YAP signaling pathway	[68]
HAND2-AS1	INO80 complex	live CSC	Promote the self-renewal of liver CSC	HAND2-AS1 combines with INO80 complex to promote the expression of BMPR1A and activate the BMP signaling	[71]
MALAT1	SRSF2	hASC	promote the Proliferation of neurons in the brain injury site of hASC	MALAT1 promotes the splicing of PKCδII by binding with SRSF2	[73]
tsRMST	Nanog and SUZ12	hESC	Inhibit the differentiation of hESC	tsRMST interacts with Nanog and SUZ12 to form a complex and inhibits the expression of Wnt5A through differentiation-related transcription factors, and inhibits non-standard Wnt pathway	[79]
Pnky	PTBP1	NSC	Inhibit the differentiation of NSC	Pnky interacts with PTBP1 to maintain the stability of its mRNA	[91, 92]
ANCR	PTBP1	hAMSC	Inhibit the differentiation of hAMSC into DE cell	ANCR binds to PTBP1, promotes the interaction between PTBP1 and ID2 mRNA and enhances the stability of ID2 mRNA	[93]
KB-1980E6.3	IGF2BP1	BCSC	Promote the stemness and proliferation of BCSC	KB-1980E6.3 binds to IGF2BP1 to form a complex under the induction of HIF-1α to recognize and enhance the stability of c-myc mRNA	[94]

Although some progress has been made in the research field of lncRNAs, and RBPs and in-depth understanding has been obtained in the basic research on lncRNAs and their RBPs in the self-renewal and differentiation of stem cells, their regulatory mechanisms are complex and there are still many molecular mechanisms that have not been clearly elucidated, and the roles played by lncRNAs and their RBPs in stem cells deserve further investigation. To date, many achievements and breakthroughs have been obtained about the other ncRNAs and their RBPs involved in stem cell self-renewal and differentiation. For example, miR-342-5p inhibits the differentiation of NSCs/intermediate neural progenitor cells (INPCs) into astrocytes, likely mediated by directly targeting site 1 and site 2 in the 3’UTR of GFAP mRNA and inhibiting GFAP expression [103]. Up-regulation of miR-145 is essential for normal neuronal differentiation. During NSCs differentiation, miR-145 interacts with Sox2 and lin28 mRNAs to down-regulate their expression and increase let-7 levels, thereby facilitating the differentiation NSCs into neurons [104]. CircRNAs play crucial roles in the initiation and development of diseases and gain significant attention. Several circRNAs are enriched in undifferentiated hESCs, such as circBIRC6, circCORO1C and circFOXP1, which are functionally associated with stemness. They act as “sponges” to recruit RBPs to monitor the stem cell self-renewal and differentiation. Knockdown of circular RNA H19 induces adipogenic differentiation of human adipose-derived stem cells (hADSCs) via interacting with PTBP1 [105]. Basic research is an important cornerstone to promote the development of clinical medicine and scientific civilization. The above research results bring new thinking and direction for future research on the biological functions of stem cells. However, the research of other ncRNAs such as tRNA and snoRNA., and their RBPs in stem cells needs to be further explored. Therefore, studying the effects of different RNAs combined with their RBPs on stem cell functions and in-depth investigation on the regulatory mechanisms can deepen our understanding of the self-renewal and differentiation of stem cells, and of the occurrence and development of relevant diseases, provide new ideas and strategies for disease prevention, diagnosis and treatment, and offer references for applications of pluripotent stem cells in clinical biomedical research.

## Data Availability

Not applicable.

## Abbreviations

LncRNAs: Long noncoding RNAs
RBP: RNA-binding proteins
ncRNAs: Noncoding RNAs
ESCs: Embryonic stem cells
ASCs: Adult stem cells
iPSCs: Induced pluripotent stem cells
NSCs: Neural stem cells
MSCs: Mesenchymal stem cells
HSCs: Hematopoietic stem cells
RSCs: Reproductive stem cells
TSCs: Totipotent stem cells
PSCs: Pluripotent stem cells
MSCs: Multipotent stem cells
USCs: Unipotent stem cells
ncRNAs: Noncoding RNAs
hESCs: Human ESCs
miRNAs: MicroRNAs
siRNAs: Short interfering RNAs
piRNAs: Piwi-interacting RNAs
snoRNAs: Small nucleolar RNAs
shRNAs: Short hairpin RNAs
circRNAs: Circular RNAs
Gas5: Growth arrest specific 5
ceRNAs: Competitive endogenous RNAs
SATB2: Special AT-rich sequence-binding protein 2
BMSCs: Bone marrow mesenchymal stem cells
MALAT1: Metastasis-associated lung adenocarcinoma transcript 1
RNP: Ribonucleoprotein
RBDs: RNA-binding domains
STAT3: Signal transducer and activator of transcription 3
PDLSCs: Periodontal ligament stem cells
Lin28A: Lin28 homolog A
Lin28B: Lin28 homolog B
TUG1: Taurine up-regulated gene 1
PRC2: Polycomb inhibitory complex 2
EZH1: Enhancer of zeste homolog 1
EZH2: Enhancer of zeste homolog 2
EED: Embryonic ectoderm development
SUZ12: Suppressor of zeste 12
SAM: S-adenosine-l-methionine
H3K27: Lys27 of histone H3
HMTase: Histone methyltransferase
hMSCs: Human mesenchymal stem cells
MEG3: Maternally expressed 3
hDFSCs: Human dental follicle stem cells
mESCs: Mouse ESCs
Oct4P4: Oct4 pseudogene 4
LincRNAs: Large-intergenic noncoding RNAs
PRESS1: P53-regulated and ESC-associated 1
BCSCs: Bladder cancer stem cells
hnRNPK: Heterogeneous ribonucleoprotein K
FGF: Fibroblast growth factor
ERK: Extracellular signal-regulated kinase
BMP: Bone morphogenetic protein
Trincrl: Trim71 interacting long noncoding RNA 1
Trim71: Trincr1 binds to tripartite motif 71
SHCBP1: SHC binding and spindle associated 1
LIF: Leukemia inhibitory factor
PPIE: Peptidylprolyl isomerase E
β-TrCP: Beta-transducin repeat containing E3 ubiquitin protein
SWI/SNF: Switch/sucrose nonfermentable
TCF7: T cell factor 7
Liver CSCs: Liver cancer stem cells
YAP: Yes-associated protein
hDPSCs: Human dental pulp stem cells
LATS1: Large tumor suppressor 1
HAND2-AS1: Heart and neural crest derivatives expressed 2-antisense RNA 1
BMPR1A: Bone morphogenetic protein receptor 1A
HCC: Hepatocellular carcinoma
PKCδ: Protein kinase C δ
hASCs: Human adipose stem cells
SRSF2: Splicing factor serine-rich arginine splicing factor 2
IGF2BP1: Insulin-like growth factor 2 mRNA-binding protein 1
CRD: Coding region instability determinant
BCSCs: Breast cancer stem cells
tsRMST: Trans-spliced noncoding RNA RMST
IPA: Ingenious Pathway Analysis
hnRNPs: Heterogeneous nuclear ribonucleoproteins
PTBP1: Polypyrimidine tract binding protein 1
ANCR: Anti-differentiation noncoding RNA
ID2: Inhibitor of DNA binding 2
hAMSCs: Human adipose-derived mesenchymal stem cells
DECs: Definitive endoderm cells
nESCs: Naive embryonic stem cells
hPDLCs: Human periodontal ligament cells
TOBF1: Transient octamer binding factor 1
FOXD3: Forkhead box D3
G3BP1: Ras-GTase-activating protein SH3 domain binding protein 1
Ras-GAP: Ras GTPase activating protein
INPCs: Intermediate neural progenitor cells
hADSCs: Human adipose-derived stem cells
